# Incidence of prolonged transitional neonatal hypoglycemia and associated factors among neonatal admissions in South Gondar public hospitals, North-Central Ethiopia: a prospective cross-sectional study

**DOI:** 10.3389/fped.2024.1381867

**Published:** 2024-10-24

**Authors:** Solomon Demis Kebede, Tigabu Munye Aytenew, Kindu Agmas, Worku Necho Asferie, Natnael Moges

**Affiliations:** ^1^ Department of Pediatrics and Neonatal Nursing, Debre Tabor University, Debre Tabor, Ethiopia; ^2^ Department of Nursing, Debre Tabor University, Debre Tabor, Ethiopia; ^3^ Department of Pediatrics and Child Health, Debre Tabor Comprehensive Specialized Hospital, Debre Tabor, Ethiopia

**Keywords:** prolonged hypoglycemia, neonatal hypoglycemia, newborn hypoglycemia, abnormal transition, persistent hypoglycemia of infant, South Gondar public hospitals

## Abstract

**Background:**

Unlike in developed countries, neonatal morbidity and mortality are the leading challenges associated with easily preventable and treatable disorders during the neonatal period in low- and middle-income countries. However, evidence-based data about prolonged transitional hypoglycemia and associated factors are highly limited in Ethiopia and resource-limited countries.

**Methods:**

An institution-based prospective cross-sectional study was conducted at public hospitals in South Gondar in neonatal intensive care units (NICUs). The data were entered and analyzed using SPSS version 23. Descriptive statistics were used to summarize maternal characteristics. Multivariate binary logistic regression at a *p* value <0.05 was used.

**Results:**

A total of 400 neonates, admitted to NICUs in public hospitals within 48–72 h of birth between October 2, 2021, and June 30, 2022, were included in the study. The incidence of prolonged transitional neonatal hypoglycemia (PTHG) was 23.5% (19.3%–28%). The factors associated with PTHG were hypothermia (AOR = 4.41; 95% CI = 2.72–10.92), preterm birth (AOR = 3.5; 95% CI = 1.69–11.97), perinatal asphyxia (AOR = 2.5; 95% CI = 1.34–9.67), and pathological jaundice (AOR = 2.3; 95% CI = 1.21–10.34). In contrast, spontaneous vaginal delivery (SVD) was a protective factor (AOR = 0.72; 95% CI = 0.35–0.88).

**Conclusions:**

The incidence of (PTHG) was nearly one-fifth. Factors increasing the risk of PTHG were hypothermia, preterm birth, perinatal asphyxia (PNA), early onset of sepsis (EONS), and pathological jaundice. Spontaneous vaginal delivery (SVD) was also a protective factor. Preventing neonatal hypothermia was the main measure used to reduce PTHG in the study area. Special attention could be given to neonates with prematurity, early onset neonatal sepsis (EONS), birth asphyxia, and pathological jaundice, as monitoring their RBS could lead to a significant change in reducing PTHG.

## Background

Deviations from the normal transition can occur as a result of a wide range of factors predisposing individuals to neonatal hypoglycemia. Thus, the main mechanisms by which these factors result in hypoglycemia include disruption of gluconeogenesis decreased alternate fuel production, increased glucose demand, and failure to receive or absorb nutrients (1–4).

However, in instances of prolonged hypoglycemia, the liver produces ketone bodies, which are partly able to produce fuel for the brain's metabolism; lactate may also be used as fuel, which is associated with metabolic acidosis, which in turn affects central nervous system symptoms (5–7).

The variability of neonatal blood glucose levels and responses and lack of outcome data are the challenges of defining neonatal hypoglycemia consistently across various populations. Ideally, clinically significant neonatal hypoglycemia is defined as the blood glucose concentration at which an intervention should be initiated to avoid significant morbidity, especially neurologic defect (1–3, 8, 9).

Neonatal hypoglycemia is still a severe disease associated with an important risk of rapidly developing severe mental visual deficit, intellectual disability, and epilepsy. According to the Women and Child Health section of the WHO, preterm and small for gestational age (SGA) neonates exhibit a constrained blood glucose regulatory mechanism for hypoglycemia. Thus, detection and treatment of these groups of neonates are targeted to reduce neonatal death, mainly in low-resource countries (10–13).

A random blood glucose level (RBS) less than 47 mg/dl (2.6 mmol/L), if present after 48 h during the first two months of life, was significantly correlated with acute seizure activity, abnormal motor manifestations, and impaired intellectual performance at 18 months of age (1, 3).

Despite progress over the past two decades, the age distribution of the mortality of children and young adolescents shows that the highest risk of death occurs during the neonatal period (the first 28 days of life), during which preventable causes accounted for 2.5 million out of 6.2 million deaths of children and young adolescents in 2018 alone. The neonatal mortality rate was estimated to be 18 deaths/1,000 live births globally. The probability of dying after the first month and before reaching age 1 was 11 per 1,000 live births (3, 14).

Prolonged neonatal hypoglycemia (PTHG) is an under-recognized cause of morbidity and mortality. In some studies, the incidence of PTHG varies due to different factors; as a result, it cannot be estimated easily by findings from other countries (1, 15–18).

The increased prevalence of prematurity and low birth weight make such studies vital to the formulation of recommendations in the most significant population of neonates to reduce neonatal mortality in specific and under-five children in general. According to the Ethiopian Demographic Health Survey (EDHS), Ethiopia has one of the highest neonatal mortality rates in the world (29 per 1,000 live births), which represents poor progress in reducing the rate of neonatal mortality from 2000 to 2016 according to the 2016 EDHS. Decreasing preventable neonatal death is an essential public health concern for resource-limited countries, particularly Ethiopia (15, 19–22).

Although a preprint of this manuscript has previously been published in Research Square (19), there is no known documented evidence of the incidence of PTHG in Ethiopia specifically or in Africa at large. The incidence of PTHG in Ethiopia is not clearly understood or documented. Hence, this study identified this important public health problem in the neonatal period. The authors also identified the factors associated with PTHG, which in turn contributes to neonatal mortality.

## Methods

### Study design and setting

This was an institutional-based prospective cross-sectional study of admitted neonates at South Gondar public hospitals in neonatal intensive care units (NICUs) from 02 October 2021 to 30 June 2022. In the study area, South Gondar Zone, there are eight public hospitals, namely, Debre Tabor Specialized and Comprehensive Hospital, Addis Zemen District Hospital, Ebnat District Hospital, Mekane-Eyesus District Hospital, Andabet District Hospital, Wogeda District Hospital, Nefas Mewucha District Hospital, and Tach Gayint District Hospital, through which neonatal care services were delivered. There are maternal and child health (MCH), inpatient, outpatient (OPD), emergency, and referral services in all the *woredas* in which the data were collected.

In addition to the zonal-level health care delivery system, the *woreda* level provides services in its catchment area. There is at least one health center and one district hospital in each *woreda.* At each district hospital, there is at least one tertiary neonatal nurse (a Bsc neonatal nurse professional) providing neonatal care to general practitioners (GPs) in the neonatal intensive care unit (NICU).

Debre Tabor Comprehensive and Specialized Hospital at the zonal level and the remaining seven public district hospitals were clustered in the zone and each of the *woreda*, respectively, within their catchment area. The data collection procedure was conducted at each cluster proportional to size (PPS) based on the annual neonatal case flow data. All neonates aged 48–72 h were selected from among all admitted neonates in this study.

### Sample size measurements and data collection procedures

The single population proportion was calculated with the following assumptions: 95% confidence interval (CI), 5% margin of error, and 50% incidence of PHG.

$$n=\frac{\;{\left(Z\;\alpha /2\right)}^{2}P(1-P)}{d^{2}}$$

Where

*N* = minimum sample size required for the study

*Z* = standard normal distribution (*Z* = 1.96) with a confidence interval of 95%

*P* = proportion of the PTHG (0.5)

*d* = is a tolerable margin of error (*d* = 0.05). The sample size calculation to determine the incidence of PHG by using the above formula was 384.

By adding a 10% nonresponse rate, the final sample size (*N*) was 422.

### Study participants and sampling method

A cluster sampling technique proportional to size (PPS) was employed among admitted neonates in the intensive care unit (NICU) at public hospitals in South Gondar. Before a maternal interview, the data collectors ascertained the case in the neonatal intensive care unit (NICU) and extracted information from the checklist for a neonatal biophysical profile, such as cause of admission, gestational age, mode of delivery, presentation, rupture of membranes, duration of labor and other information from the labor delivery room referral report form.

The data were extracted (collected) by BSc neonatal nurses and health information technicians or delivery ward coordinators for verification of the data at each health facility. The data collectors and supervisors had one day of training from a principal investigator about the tool for data collection and abstraction.

### Data collection tool and procedures

The random glucose level of the neonates (RBS) was measured by using a glucometer device with blood glucose test strips, and the selected study participants were tested for hypoglycemia by piercing the outer part of their heel as per routine measurement procedures in each hospital. The measurements were taken twice a day at 6 AM in the morning and at 6 PM in the afternoon within 48–72 h of neonatal age. Two RBS measurements, morning and afternoon, were subsequently recorded on the data collection form.

Clinical information such as gestational age (GA), mode of delivery, duration of labor, and causes of admission, such as jaundice, prenatal asphyxia (PNA), and early-onset neonatal sepsis (EONS), was collected from individual neonates (Neonatal Chart). The maternal interview was conducted after taking her neonatal RBS record in the mother's waiting room at her convenient time and with her verbal consent.

The tool was formulated after referring to various pieces of literature. The interview questionnaire consisted of information on socio-demographic characteristics; pregnancy and obstetric factors; medical and other factors; and neonatal profiles from labor delivery referral forms and admission history (4, 12, 16, 17, 21–23).

### Data quality control plan

Data accuracy, completeness, and timeliness were checked by the following activities. The training of the data collectors and supervisors for one day regarding the objective of the study, the data collection tool, and the methods of data collection, checking the completeness of the data collection tool, and maintaining confidentiality by coding and limiting access to the collected pieces of information was completed.

The data collection tool was pretested by Debre Tabor Comprehensive Specialized Hospital. A pretest was administered, questions that were not clear were corrected, and a discussion was held with the data collectors and supervisors on the problems they encountered in completing the checklist. The data were checked for completeness before data entry into epi version 4.2. Proper coding of the data and categorization of outcome variables into persistent hypoglycemia and not were maintained for the quality of the data to be analyzed. Similarly, temperature data were categorized as hypothermic or normothermic according to the operationalized definition used in this study. Double data entry was performed for validity, and the data were compared to the original data. Simple frequencies and cross-tabulations were used to check missing and outlier variables, which were cross-checked with hard copies of the collected data.

### Outcome variable measurement

Prolonged transitional hypoglycemia (PTHG) was defined as a random blood glucose level (RBS) < 47 mg/dl measured by a tool labeled as Gmate-Voice at 6 AM on the morning and at 6 PM in the afternoon for any gestational age within 48–72 h of life after delivery (1, 10, 24, 25).

Hyperglycemia: The latest record of a random blood sugar level (RBS) > 125 mg/dl was obtained at 6 AM in the morning and at 6 PM in the afternoon within 48–72 h after delivery (25).

Normglycemic status: The latest record of a random blood glucose level (RBS) of 47 mg/dl–125 mg/dl was obtained at 6 AM in the morning and at 6 PM in the afternoon within 48–72 h after delivery (24, 25).

Hypothermia was defined as the most recent measurement of an auxiliary body temperature within 48–72 h of age, which was less than 36.5°C (23).

Neonate: Newborns within the first 28 days of age after live birth with a viable gestational age (GA > 30 weeks) were included in this study.

### Data processing and analysis

The data were entered and analyzed using EpiData 4.2 and SPSS version 23. Descriptive analysis was performed by computing proportions and summary statistics. The data are presented as simple frequencies, summary measures, tables, and figures. Missing values were analyzed by using multiple imputation techniques.

Binary logistic regression was used to analyze the outcome variables. Bivariate and multivariate analyses were performed to determine the associations between the outcome variables and each independent variable. The assumptions for binary logistic regression were checked. The goodness of fit was tested by the Hosmer–Lemeshow test and Omnibus test. All variables with *P* < 0.25 in the bivariate analysis were included in the final model of multivariate analysis to control for all possible confounders, and the variables were selected by the method.

The direction and strength of the associations were measured by odds ratios (ORs) with 95% confidence intervals (CIs). The adjusted odds ratio (OR) and 95% confidence interval (CI) were estimated to identify factors associated with persistent neonatal hypoglycemia via multivariate analysis via logistic regression. In this study, a *P* value < 0.05 was considered to indicate statistical significance.

## Results

### Maternal socio-demographic characteristics

Out of 422 mothers whose neonates were admitted to neonatal intensive care units in the study areas fulfilled the criteria for enrollment in the study. Approximately 284 (67.30%) of the participants were aged 20–34 years, whereas the majority of the mothers were married [392 (92.90%)] (Table 1).

**Table 1:** Maternal socio-demographic characteristics of prolonged transitional hypoglycemia and associated factors among admitted neonates at South Gondar public hospitals, 2022.

Characteristics		Frequency	Percentage (%)
Age of mothers	<20 years	16	3.71
	20–34 years	284	67.30
	≥35 years	122	28.90
Marital status	Married	392	92.90%
	Not married	28	6.63%
	Others	2	0.47
Residence	Rural	219	51.80%
	Urban	203	48.20%
Educational status	No formal education	171	40.50%
	Can read and write	91	21.60%
	Primary school	41	9.70%
	Secondary school	56	13.30%
	College and above	63	14.90%
Occupation	Housewife	223	52.80%
	Employers	119	28.20%
	Others	80	19.00%

### Incidence of prolonged transitional hypoglycemia

The median and range of random blood glucose levels at 48–72 h of age were 62 gm/dl, and the RBS ranged from 35 gm/dl to a high level, which was not recorded by a glucometer. For instance, high RBS levels can reach more than 250 mg/dl.

**Figure 1:**
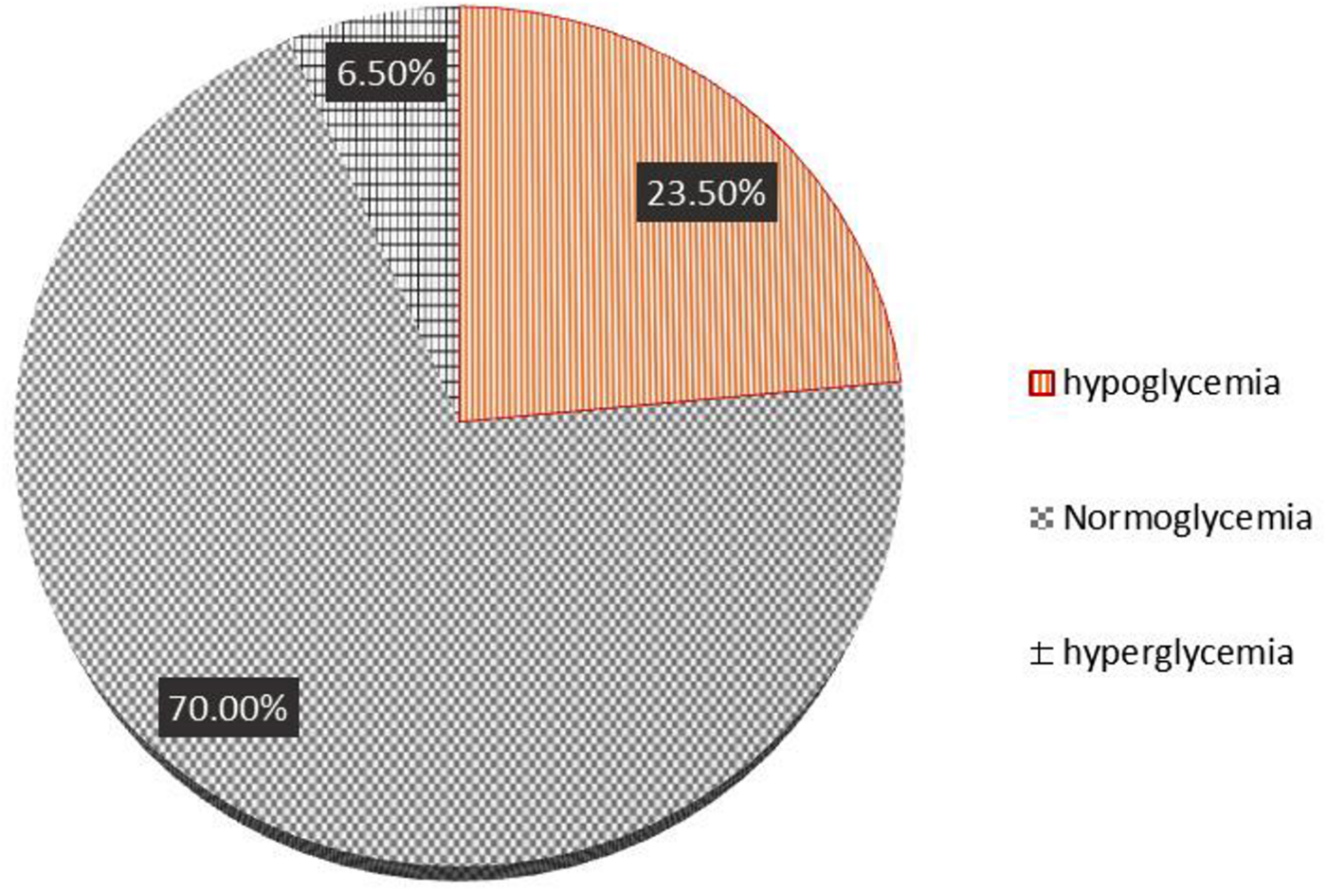
Incidence of persistent hypoglycemia.

The incidence of persistent hypoglycemia (PTHG) was 94 (23.5%) (19.3%–28%), 280 (70%) neonates were in a normoglycemic state, and approximately 26 (6.50%) were hyperglycemic. Figure 1 shows the incidence of persistent neonatal hypoglycemia.

### Pregnancy and obstetric characteristics

Approximately 218 (56.41%) mothers had a duration of labor between 1 and 12 h, and the majority of them delivered their current pregnancy through SVD, accounting for 96 (70.26%) of the mothers. The majority of the patients had a rupture of membrane (ROM) 12 h or less [365 (93.84%) of the patients] (Table 2).

**Table 2:** Pregnancy and obstetrics characteristics of prolonged transitional hypoglycemia and associated factors among admitted neonates in South Gondar public hospitals, 2022.

Variables	Frequency (*n* = 422)	Percent (%)
Duration of labor (DOL)	*N* = 385	Percent
1–12 h	218	56.41
12–24 h	148	38.46
Above 24 h	19	5.13
Mode of delivery	*N* = 422	Percent (%)
SVD	296	70.26
Forceps	33	7.69
Vacuum	39	9.23
C/S	54	12.82
Presentation at labor	*N* = 389	Percent (%)
Cephalic	315	81.02
Breech	66	16.92
Others	8	2.06
Gestational age at birth	*N* = 389	Percent (%)
28–32 weeks	45	10.80
32–36 weeks	119	28.20
37–42 weeks	232	54.80
Above 42 weeks	13	3.10
Unknown	13	3.10
ANC visit during pregnancy	*N* = 422	Percent (%)
Yes	385	91.28
No	37	8.72
Duration of rupture of membrane (ROM)	*N* = 389	Percent (%)
≤12 h	365	93.84
>12 h	24	6.16

### Maternal health problems during pregnancy

**Figure 2:**
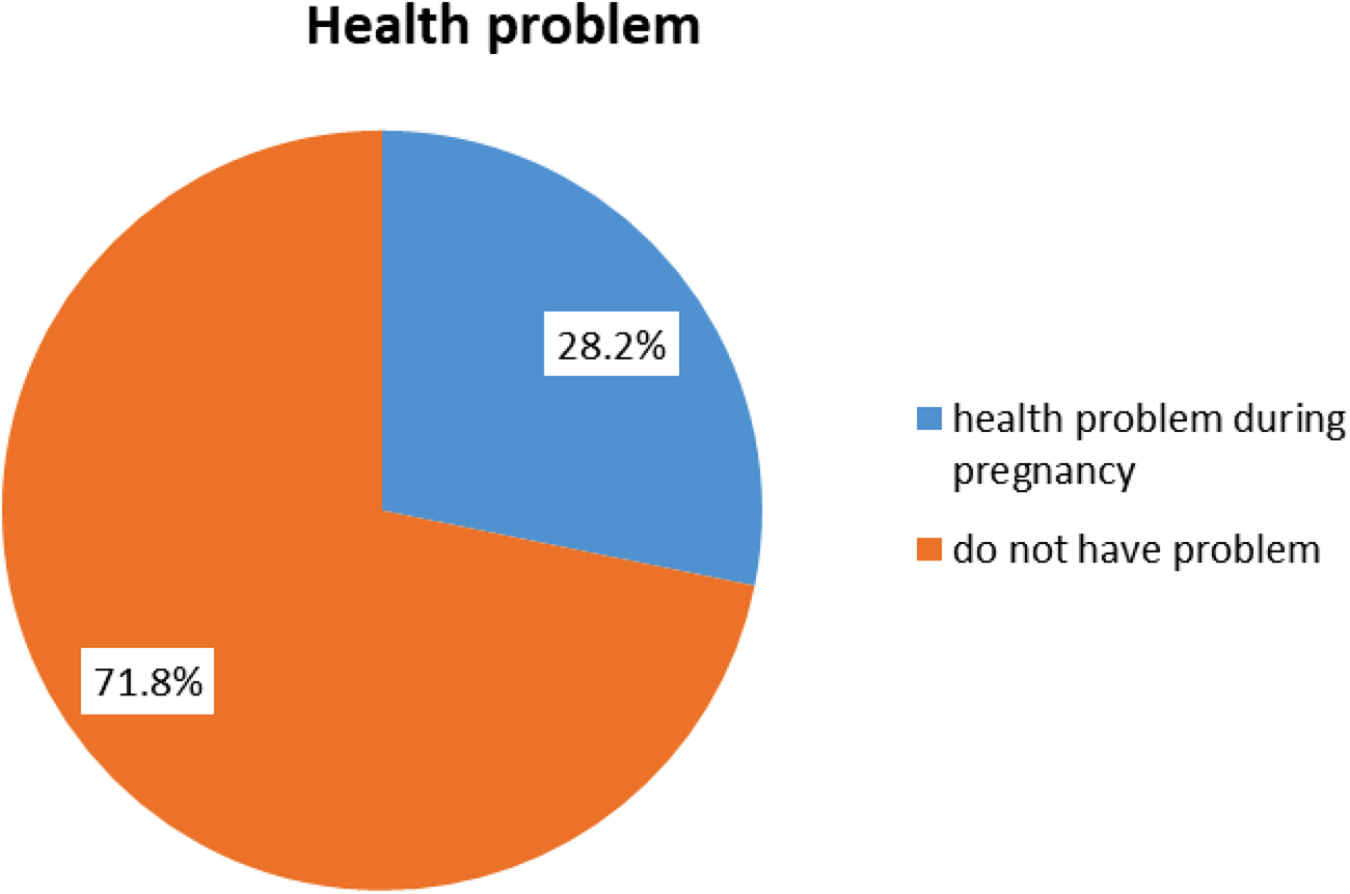
Maternal health problems during pregnancy.

The majority of the 303 (71.8%) women did not have health problems during pregnancy, while the remaining 119 (28.2%) had different health problems during their latest pregnancy. Figure 2 shows maternal health problems during pregnancy.

### Types of health problems during pregnancy

**Figure 3:**
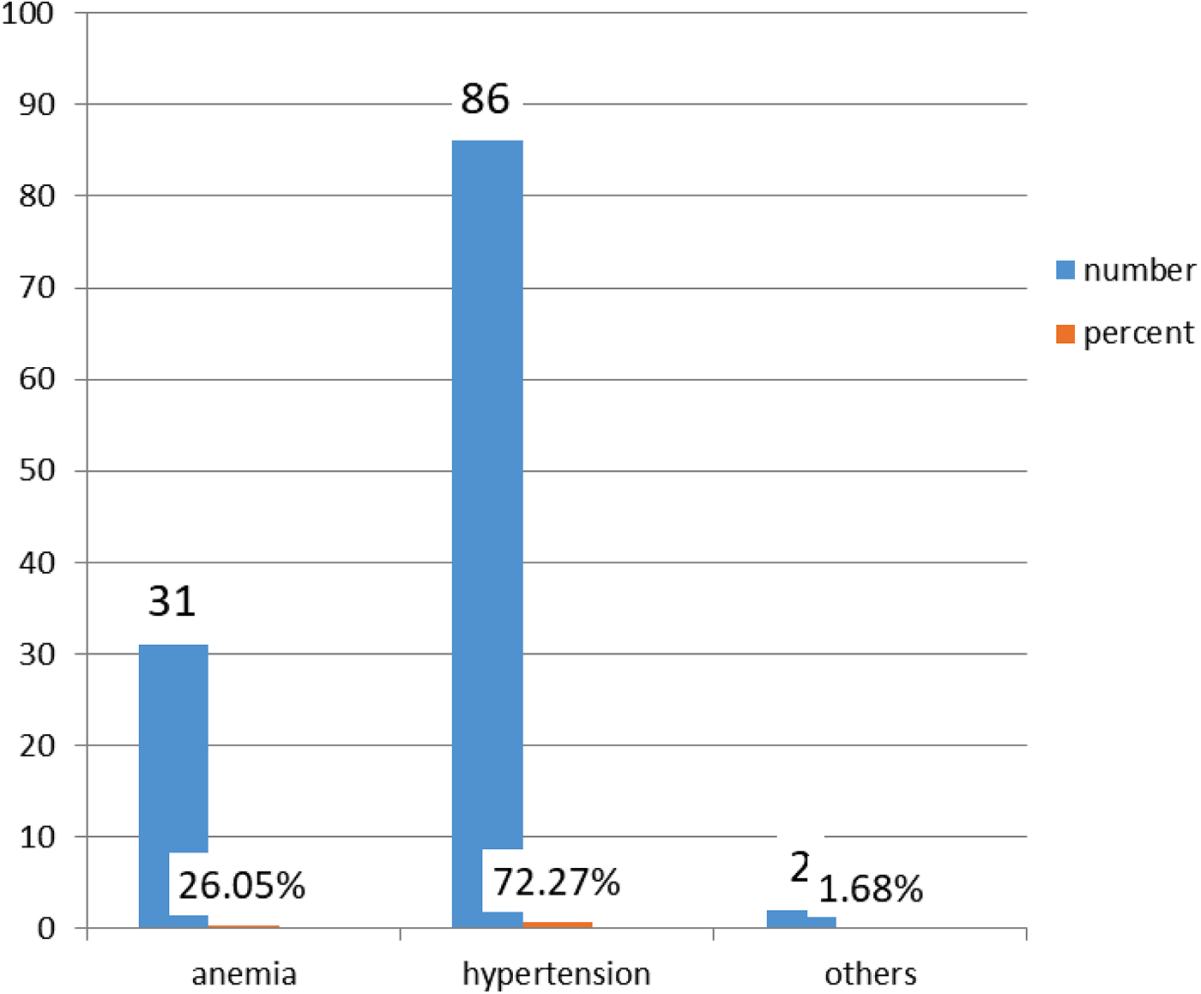
Types of maternal pregnancy health problems.

Among the 119 women who had a health problem, 86 (72.27%) had hypertensive disorders during pregnancy and 2 had other disorders (1.68%). Figure 3 shows the types of maternal pregnancy health problems.

### Neonatal profiles at admission

The most common cause of admission was hypothermia (100, 25%), the least common cause was other causes (20, 5%), and the majority of neonates were born at term (*n* = 232, 54.80%). The number of very-low-birth-weight neonates was the lowest among the groups, accounting for 10 (2.56%) (Table 3).

**Table 3:** Neonatal profile for incidence and associated factors of prolonged transitional neonatal hypoglycemia among admitted neonates in the NICU at South Gondar public hospitals, 2022.

Cause of admission	Frequency	Percent (%)
Hypothermia	100	25.00
PNA	67	16.80
Sepsis (EONS)	48	12.00
Jaundice	89	22.20
SGA	32	8.00
RDS	22	5.50
Twin	22	5.50
Others	20	5.00
Presentation at labor	Frequency	Percent (%)
Cephalic	315	81.02
Breach	66	16.92
Others	8	2.06
Gestational age at birth	Frequency	Percent (%)
28–32 weeks	45	10.80
32–36 weeks	119	28.20
37–42 weeks	232	54.80
Above 42 weeks	13	3.10
Unknown	13	3.10
Birth weight	Frequency	Percent (%)
VLBW	10	2.56
LBW	209	53.85
NBW	167	43.59

### Factors associated with prolonged transitional hypoglycemia

Binary logistic regression was used to identify factors associated with PTHG. There was no marked variation according to the number of deliveries, intentions to current birth, pregnancy disorders, educational status, or maternal age group, even though these factors were associated with each other according to bivariate analysis.

Preterm birth, hypothermia, perinatal asphyxia (PNA), early onset of sepsis (EONS), and clinical pathological jaundice were risk factors for PTHG, while spontaneous vaginal delivery (SVD) was protective.

Neonates with hypothermia were 4 times more likely to develop PTHG than were those with normothermic hypothermia (AOR = 4.41; 95% CI = 2.72–10.92); preterm birth was 3.5 times more likely to be associated with PTHG than was term birth (AOR = 3.5; 95% CI = 1.69–11.97); perinatal asphyxia was 2.5 times more likely to lead to PTHG than was no asphyxia (AOR = 2.5; 95% CI = 1.34–9.67); and neonates with pathological jaundice were almost 2 times more likely to have PTHG than were those with no jaundice problems (AOR = 2.3; 95% CI = 1.21–10.34).

In contrast, spontaneous vaginal delivery (SVD) was protective and was 72% less likely to develop vaginal delivery than was cesarean section delivery (AOR = 0.72; 95% CI = 0.35–0.88) (Table 4).

**Table 4:** Factors associated with the incidence of PTHG among admitted neonates in the NICU at South Gondar public hospitals, 2022.

Variables	Category	COR	AOR
Mode of delivery	SVD	1.76 (0.35–8.82)	0.72 (0.35–0.88)*
	Vacuum	4.40 (1.32–14.64)	0.44 (0.07–2.82)
	CS	1	1
Gestational age at birth (in weeks)	30–36	3.88 (1.23–12.24)	3.5 (1.69–11.97)**
	37–42	1	**1**
Current delivery number	Multiple	5.20 (2.60–15.45)	1.67 (0.86–3.80)
	Singleton	1	1
Rectal temperature	Hypothermia	2.44 (1.02–5.84)	4.41 (2.72–10.92)**
	No/mild hypothermia	1	1
The intention of the current pregnancy	Wanted and supported	2.45 (1.23–16.78)	1.23 (0.50–6.40)
	Unwanted and supported	3.60 (2.86–9.20)	2.46 (0.88–11.20)
	Unwanted and unsupported	1	1
Perinatal asphyxia	Yes	3.25 (2.0–8.5)	2.50 (1.34–9.67)**
	No	1	1
Disorders during pregnancy	Hypertension	5.20 (3.40–17.30)	2.15 (0.70–11.30)
	Anemia	2.33 (1.10–13.64)	3.23 (0.86–14.20)
	Others	1	1
Early onset neonatal sepsis (EONS)	Yes	3.20 (2.0–8.72)	2.0 (1.10–9.50)*
	No	1	1
Maternal educational status	No formal education	5 (1.3–17.37)	1.17 (0.47–5.56)
	Can read and write	1.90 (1.23–21.40)	3.99 (0.19–5.0)
	Primary school	0.21 (0.23–1.93)	0.58 (0.03–8.62)
	Secondary school	0.15 (0.01–1.37)	0.16 (0.01–2.27)
	College and above	1	1
Clinical jaundice	Yes	4.21 (2.30–8.76)	2.30 (1.21–10.34)**
	No	1	1
Maternal age in years	<20	3.46 (0.90–2.12)	2.56 (0.43–2.56)
	20–34	2.30 (1.10–7.55)	1.23(0.67–3.88)
	>=35	1	1

A variable significant at *p* < 0.2 according to the bi-variable analysis.

* Significant at *p* ≤ 0.05.

** Significant at *p* ≤ 0.001, 1 = reference.

## Discussion

### Incidence of prolonged transitional neonatal hypoglycemia (PTHG)

The incidence of PTHG was 94 (23.5%; 19.3%–28%) in this study. According to the literature, under diagnosing or misdiagnosing neonatal hypoglycemia can lead to neonatal morbidity and mortality, which can be attributed to the aggravating factors of hypoglycemia. There have been underreporting or nearly absent studies about neonatal hypoglycemia globally and in Africa about persistent hypoglycemia.

The incidence estimates of hypoglycemia in the newborn depend both on the definition of the condition and the methods by which blood glucose concentrations are measured as well as the cutoff point of time for taking the measurement.

Studies Globally, conducted in Royal Alexandra Hospital, Canada, Denver Colorado, Waikato District Health Board, Hamilton, New Zealand, according to the American Association of Pediatrics in the entire population estimate, Boston, USA, showed that the incidence of neonatal hypoglycemia within 48 h of age ranged from 30%–63%, which was higher than that in this study (14, 16–19).

Unpublished findings indicated that 25% of neonatal hypoglycemic agents were diagnosed at St. Paul Millennium Hospital Medical College in Ethiopia (2).

The variation might be because the studies used transient hypoglycemia within 48 h after delivery, which in turn consisted of physiological hypoglycemia that subsequently returned to the normal glycemic state after the physiological process (48–72 h). This discrepancy might arise from the sample size variation and some clinical setup variation found among studies.

According to other reports, for early neonatal hypoglycemia, which was screened in Turkey, Denver Colorado, and Israel, the incidence of hypoglycemia was 12%–17%, and 12.1%, which was lower than that in this study (20, 26, 27).

This variation might arise from the definition of the condition and the methods by which blood glucose concentrations are measured as well as the cutoff point of time for taking the measurement.

### Factors associated with PTHG

Preterm birth, hypothermia, perinatal asphyxia (PNA), early onset of sepsis (EONS), and pathological jaundice were factors associated with PTHG, while spontaneous vaginal delivery (SVD) was protective.

Compared with normothermic neonates, neonates with hypothermia were 4 times more likely to develop PTHG; preterm birth was 3.5 times more likely to be associated with; and perinatal asphyxia was 2.5 times more likely to develop than it was in noasphyxia. Neonates with pathological jaundice were almost 2 times more likely to be PTHG agents than were those with no jaundice.

There are various risk factors for neonatal hypoglycemia, as reported in the literature, depending on the population and area of study, making it difficult to use these data consistently worldwide.

In line with these findings, in Israel and West Virginia University Hospital, hypothermia, low birth weight (<2,500 g), prematurity, small for gestational age (SGA), and perinatal asphyxia were found to be independent predictors of neonatal hypoglycemia (2, 13, 27).

This might be because hypothermia causes an increased demand for glucose metabolism for thermoregulation in addition to the demand for an extra uterine adaptation process. When the neonate is cold, he or she uses more glycogen to stay warm. Then, he utilized his glucose stores to stay warm, his blood sugar concentration decreased, and he became hypoglycemic and hypothermic.

Similarly, premature individuals are predisposed to developing hypoglycemia and associated complications due to their limited glycogen and fat stores, inability to generate new glucose via gluconeogenesis pathways, increased metabolic demand due to relatively greater brain size, and inability to mount a counter regulatory response to hypoglycemia.

In Ethiopia, the risk factors associated with neonatal hypoglycemia were birth weight, duration of labor, maternal age, duration of feeding initiation, hypothermia, and respiratory distress syndrome, and some of the factors leading to hypoglycemia were identified as consistent with the findings of this study (2).

These factors associated with hypoglycemia increase the risk of developing hypoglycemia because hypothermia, low birth weight, and respiratory distress syndrome affect the normal life of neonates from intrauterine to extra uterine life.

Conversely, this study revealed that spontaneous vaginal delivery (SVD) was protective and was 72% less likely to develop than cesarean section delivery was.

Preventing and treating PTHG is required because it leads to neurological damage, intellectual disability, epilepsy, personality disorders, impaired cardiac performance, and muscle weakness (23–29).

### Strength and limitation

Preventing and treating PTHG prevents neurological damage, intellectual disability, epilepsy, personality disorders, impaired cardiac performance, and muscle weakness attributed to PTHG during the neonatal period. It highlights the consequences of adequate postnatal care in preventing persistent neonatal hypoglycemia. However, because of the cross-sectional nature of the study, we could not determine the causal relationships.

## Conclusions

The PTHG was lower than that reported in previous studies, while factors increasing the risk of PTHG were preterm birth, hypothermia, perinatal asphyxia (PNA), early onset of sepsis (EONS), and clinical pathological jaundice. Spontaneous vaginal delivery (SVD) was found to be a protective factor. Preventing neonatal hypothermia is the main measure used to reduce the PTHG in the study area as well as in resource-limited areas in Ethiopia. Preventing neonatal hypothermia was the main measure used to reduce PTHG in the study area. Close follow-up of neonates with prematurity, early onset neonatal sepsis (EONS), birth asphyxia, and pathological jaundice due to hypoglycemia could lead to a significant change in reducing PTHG.

## Data Availability

The raw data supporting the conclusions of this article will be made available by the authors, without undue reservation.
